# Accounting for Impact? The Journal Impact Factor and the Making of Biomedical Research in the Netherlands

**DOI:** 10.1007/s11024-015-9274-5

**Published:** 2015-05-31

**Authors:** Alexander Rushforth, Sarah de Rijcke

**Affiliations:** Center for Science and Technology Studies (CWTS), Leiden University, P.O. Box 905, 2300 AX Leiden, The Netherlands

**Keywords:** Journal Impact Factor, Biomedicine, Performance indicators, Bibliometrics, Research evaluation

## Abstract

The range and types of performance metrics has recently proliferated in academic settings, with bibliometric indicators being particularly visible examples. One field that has traditionally been hospitable towards such indicators is biomedicine. Here the relative merits of bibliometrics are widely discussed, with debates often portraying them as heroes or villains. Despite a plethora of controversies, one of the most widely used indicators in this field is said to be the Journal Impact Factor (JIF). In this article we argue that much of the current debates around researchers’ uses of the JIF in biomedicine can be classed as ‘folk theories’: explanatory accounts told among a community that seldom (if ever) get systematically checked. Such accounts rarely disclose how knowledge production itself becomes more-or-less consolidated around the JIF. Using ethnographic materials from different research sites in Dutch University Medical Centers, this article sheds new empirical and theoretical light on how performance metrics variously shape biomedical research on the ‘shop floor.’ Our detailed analysis underscores a need for further research into the constitutive effects of evaluative metrics.

## Introduction

How research evaluation shapes the actual production of knowledge is an under-explored topic in various literatures addressing academic research (Gläser and Laudel 2007; Woelert 2015). This is surprising given the wave of performance and audit measures that has swept across public institutions over the past two decades – leading commentators to claim that we now inhabit ‘audit’ and ‘evaluation’ societies (Power 1997; Dahler-Larsen 2012). One of the primary institutions subject to such transformations has been the university (Schimank 2005; Krücken et al. 2013). A feature that is often associated with these developments is the dramatic rise of quantitative performance indicators (Feller 2009; Keevers et al. 2012; Nedeva et al. 2012).

Despite an abundance of literature on research evaluation from across several social science fields, how citation-based measures interact with knowledge production rarely receives attention (Wouters 2014). Science policy studies often focus on the efficacy of contemporary research evaluation programs, including proposals for improving methods and performance indicators (c.f. Cozzens and Melkers 1997; Luukkonen 2014). Higher education studies often adopt a birds-eye perspective on formal mechanisms of assessment and national evaluation systems (Fealing 2011; Reale and Seeber 2013), for instance, conducting systematic overviews of indicators used in different national funding contexts (Geuna and Martin 2003). Studies describing the rise of ‘New Public Management’ have positioned indicators as tools used to steer academic institutions towards becoming more market-oriented organizations (Parker and Jary 1995; Willmott 2011; Leisyte and Dee 2012), albeit the overall impact of reforms on different higher education systems is often contested (Bleiklie and Michelsen 2013). Nevertheless, implications are that academics have become increasingly disciplined by quantitative, pre-defined, measurable outcomes that fulfill informational and control requirements within a neo-liberal higher education system (Sauder and Espeland 2009; Shore 2010; Burrows 2012). Although we are by no means dismissive of these concerns, they can sometimes cut across the more detailed contingencies surrounding indicator uses in different research settings. This attention to detail becomes particularly pertinent if one takes seriously the idea that indicators acquire meaning through contexts of use (Dahler-Larsen 2013).

We develop this ‘constitutive’ focus on indicators through a case study of biomedical scientists’ uses of the Journal Impact Factor (JIF) in their everyday knowledge making activities. The JIF is calculated annually by a commercial company – Thomson Reuters – based on its Journal Citation Reports. Despite its presence as a long-standing measure of journals’ citation rates, recently there have been a number of widespread denunciations of the JIF from various collectives and individuals, particularly in the biomedical field itself. Notable examples of outspoken critics include Nobel Prize-winning biologist Randy Schekman (2013) and the San Francisco Declaration on Research Assessment (DORA 2013). The arguments deployed against the JIF are quite broad-ranging, but typically include claims that it is summarily misused by researchers, can be misleading when used in a number of evaluative contexts, and that it is subject to gaming among journals. Rather than add to these critical voices per se, our aim is to retrieve the actual practices that converge around the JIF in day-to-day biomedical research. The concept of ‘folk theory’ (Rip 2006) is used here to sensitize analysis more toward members’ own theorizing and appropriation of the JIF in research contexts. For Rip, folk theories are generalizations about patterns of action which are made *in ongoing practices* and which ‘serves the purposes of the members of the various practices’ themselves (Rip 2006: 349). The folk theory concept thus helps to retrieve practices surrounding the JIF in knowledge making, rather than diagnosing or classifying general misuses, or prescribing idealized solutions. An additional sensitizing concept is to treat the JIF as a ‘judgment device’ (Karpik 2010) in knowledge making, which helps rate, rank, and order judgments hierarchically (cf. Abbott 2014). Shedding new empirical and theoretical light on how an indicator like the JIF gets incorporated into knowledge production, we hope to provide a platform for further research into the under-explored area of performance indicator uses in academic settings.

The data for this article was collected as part of a larger exploratory ethnographic study into how indicators and evaluation dynamics link to biomedical knowledge production in the Netherlands. Historically the considerable scientific and societal relevance attached to biomedicine, as well as the extensive coverage of biomedical literature in the Web of Science database has led to a certain receptiveness towards performance indicators in evaluating research in this field (De Bellis 2009; Van Eck et al. 2013). Developments in size and structure in biomedicine have also led to proliferation in quantity of literature, presenting researchers with problems of quality discrimination and ‘information overload’; factors that provided momentum to promises for adopting bibliometric solutions (Woelert 2013). Dutch University Medical Centers (UMCs) account for approximately one third of all journal articles produced in the whole of the Netherlands, and whilst not hosting the entirety of biomedical research, they nonetheless cover most of it (NFU 2008). Since the end of the 1990s, eight UMCs were formed through mergers between the respective universities’ faculties of medicine and academic medical hospitals. Research is one core activity of UMCs alongside healthcare, teaching, and valorization activities, and is appraised yearly via professional bibliometric research assessments used for external monitoring purposes – an unusual rate compared to other fields. We therefore expected that indicators would be made visible to us in these settings as observers, yet we did not know how and in what situations they would figure.

The structure of the paper is as follows: first we describe the rationale behind the methods and choice of field sites. We will then locate our contribution in respect to other literatures that have discussed researchers’ uses of indicators in general, and the JIF in particular. Here, we also discuss how the analytic concepts we deploy are relevant to these discussions and our own analysis. The findings are presented in two analytic sections. The first attends to the theme of authorship and collaboration practices in the different UMC research sites and how the JIF intersected these. This section displays scientists’ own theories about the JIF across different epistemic and organizational settings, and at particular moments within their research practices, making visible the propensity for certain work patterns and relations to harden around the JIF. In the second analytic section we report how different research sites mobilized the JIF as a judgment device in planning to submit work-in-progress manuscripts. These interactions around manuscripts brought to light scientists’ folk theories linking the JIF to reputation and targeting of journals. Finally, the implications of the comparative findings for research in sociological studies of science and higher education will be considered, which calls for a need to re-think indicators and research evaluation as actual components of academic knowledge production.

## Methods

The research in this paper draws from a project about the performative effects of bibliometric indicators on biomedical research in the Netherlands.^1^ Fieldwork was carried out at two UMCs and at three research groups within each: a molecular cell biology laboratory, a surgical oncology laboratory, and medical statistics group. Here we follow works in sociology of science (Knorr-Cetina 1999; Felt et al. 2009; Whitley et al. 2010) that posit the diversity of epistemic and organizational work practices in the sciences. The multi-sited scope allows for greater attention towards interactions between indicators and knowledge production than would be afforded by a single-sited study. Conducting fieldwork in two separate UMCs (for purposes of anonymity, hereon Institution A and B) enables a focus on how the local organizational context of UMCs shapes the dynamics of evaluation and indicator usage. In addition, we identified three broad registers of biomedical knowledge production, following institutionalized distinctions between basic, translational, and applied research at each UMC. This sampling logic is also based on an assumption that different sub-fields of biomedicine pursue quite distinct patterns of knowledge production, for instance, in terms of publication and citation practices (Opthof and Wilde 2011; Van Eck et al. 2013).

Fieldwork at Institution A took place over an 8-month period from September 2012 until April 2013, and at Institution B between April and August 2014. Our research consisted of document analysis, interviews and observations. Detailed fieldnotes were recorded of meetings, laboratory-work, presentations, and of conversations with our informants. Semi-structured interviews were held with researchers (PhD-students, post-docs and senior staff), technicians, research managers, and evaluation officers. Topics included organization of the research teams and departments, formal evaluation, uses of indicators, scientific careers, funding, and publication practices. Our data generation was complemented with document analysis of materials collected online or made available via our informants, including annual review forms, institutional performance targets, and communications such as emails. Names of all individuals and organizations have been anonymized. The materials where necessary were transcribed into electronic form and uploaded onto the Nvivo qualitative software package.

The themes we present below emerged by combining sensitivity towards existing sociological literature with emerging insights from the data. As far as possible we tried to adopt an open-ended and inductive stance. A case in point is that we had not set out to study one indicator in particular, but the role of metrics in general. Yet by zooming-in on uses of indicators in UMC research settings, we found no single indicator divided opinions and practices as much as the JIF. Its central focus in this article reflects its widespread presence in our data, which in turn can be fed-back in with ongoing debates on researchers’ uses of the JIF in interesting and original ways.

## The Journal Impact Factor in Biomedical Research Practices

Thomson Reuters publicize the JIF annually using their Journal Citation Reports (JCR), measuring the average number of citations per paper from a journal during the two years preceding the publication of the JCR. It is calculated by dividing A) the number of citations in the current year to items published in that journal in the past two years by B) the total number of citable articles in the two previous years. In a reflective account, the American chemist who first developed the indicator – Eugene Garfield – recognizes that today the indicator has become ‘utilized in most countries to evaluate institutions, scientific research, entire journals, and individual articles’ (Garfield 2003: 363). Yet many claim that the JIF has extended even further:

Nowadays it is used as a direct reflection of a journal’s prestige or quality. Journal editors and publishers communicate the values of impact factors of their journals to reading audiences. Impact factors are not only used to rank journals, but to evaluate individual scholars and research groups or departments they select for publication, even in decisions about salaries or promotion (Moed 2005: 91–92).

Within a later article Garfield remarked that uses have gone far beyond what he had originally envisaged and goes on to evaluate its contemporary influence as a ‘mixed blessing’(Garfield 2006). However, others have been less equivocal. Researchers in the field of scientometrics (dedicated to the construction and advancement of existing and new bibliometric indicators), with which Garfield is usually associated, are quite often robust in their criticisms. Much of the discourse here takes a normative stance, concerning ‘what is to be done’ with regards the ‘unintended effects’ bibliometric products come to have in an era of rapidly expanding academic audit (Weingart 2005; Van Dalen et al. 2012). In 2012, a leading journal in the field – *Scientometrics* – produced a special issue concentrating on the JIF’s *uses* and *misuses* (Braun 2012). Arguments against the JIF often cite a number of technical shortcomings (Moed and van Leeuwen 1996; Buela-Casal and Zych 2012), for instance, in claiming it a ‘faulty method’ or as ‘widely open to manipulation by journal editors and misuse by uncritical parties’ (Archambault and Larivière 2009: 635). Research managers have been identified as a group especially prone to (mis)using indicators in recent sociological accounts of academic governance and research cultures (Gläser and Laudel 2007; Woelert 2015). Social scientists are not alone in voicing their concerns. In 2013, the Nobel Prize-winning biologist Randy Schekman produced a high profile criticism of practices in biomedicine, stating that the JIF has become a performance standard now dominating research practices (Schekman 2013). In 2012, a critical list of declarations – The San Francisco Declaration on Research Evaluation (DORA) – was launched by biomedical scholars and journal editors about the role the JIF plays in academic life. The statement warns against various applications of the JIF in research assessment contexts (actual evaluation, promotion or hiring, awarding grants), and reiterated a number of ‘widely accepted’ criticisms – both ‘technical’ and ‘social’ – undermining the JIF as a scientific quality indicator. In doing so DORA calls for ‘funding agencies, academic institutions, journals, organizations that supply metrics, and individual researchers’ alike to drop the indicator (DORA 2013). Another medium in which the JIF’s influence gets discussed perennially is prestigious biomedical journals. A recent statement by then Editor-in-chief of *Journal of Cell Biology* expressed support for DORA and cites a number of ways the JIF feeds into the ‘culture’ of biomedicine:

The [J]IF is pervasive in the scientific community. Scientists refer to it casually in conversation to convince colleagues of the importance of their own papers, or they wonder how a paper ended up in “a journal with such a high Impact Factor.” Students and postdocs want to publish only in “high Impact Factor” journals, and the [J]IF is frequently used in recruitment, tenure, and granting decisions when a candidate’s past publication performance is assessed (Misteli 2013: 651).

An earlier article in the *British Medical Journal* echoes a concern voiced among journal editors on the kinds of knowledge being produced, given that maintaining ‘respectable’ JIF scores necessitates editorial practices focusing ‘more and more on citations and less and less on readers’ (Brown 2007: 561). Together these accounts capture a set of ‘folk theories’ (Rip 2006) about roles and influence of the JIF in biomedicine, which are well publicized and by implication feed-back into researchers own practices concerning the JIF. But whether such general statements and assumptions about indicators translate seamlessly into biomedical research settings, and whether they do so in similar ways for different sub-fields is a topic for our analysis, rather than a starting assumption. Thus whilst we have no particular complaints with the claim that the JIF can and/or ought to be improved methodologically, we find the above-mentioned accounts of how scientists use (or ought to use) indicators fall short of what robust empirical analysis and understanding can offer. In orienting towards this task we follow Dahler-Larsen’s (2013) suggestions that oft-heard accusations of indicator ‘misuse’ would be more productively recast for analytic purposes merely as examples of actual *use*; a particular community’s ‘failure’ to follow standards of ‘proper use’ are eschewed in favor of accounts of the actual uses of indicators by researchers; and ‘unintended consequences’ are transformed into ‘constitutive effects’ of indicator use (Dahler-Larsen 2013). Indeed, a general criticism that can be made about these above accounts is their inattention towards scientists’ *own* ‘folk theories’ of indicators. Although coined by Rip in describing nanotechnologists, his definition is useful for our purposes here:

Actors attempt to capture patterns in what is happening and be reflexive about them, so as to do better the next time. Since there is a claim that such patterns will recur… there is generalization, so one can speak of a theory… Calling it a *folk* theory implies that it evolves in ongoing practices, and serves the purposes of the members of the various practices. What characterizes folk theories is that they provide orientation for future action… They are a form of expectations, based in some experience, but not necessarily systematically checked. Their robustness derives from their being generally accepted, and thus part of a repertoire current in a group or in our culture more generally (Rip 2006: 349).

The folk theories concept is helpful here as it does not take received assumptions of the JIF as given, but rather positions its role and influence in research as a topic of analysis. Asknes & Rip’s (2009) attention towards scientists’ folk theories of citations would suggest notions like ‘amateur bibliometrics’ or ‘uncritical parties’ may be somewhat dismissive. Indeed, some have flagged new forms of ‘vernacular’ knowledge in the domain of evaluative bibliometrics as potentially perturbing earlier kinds of separations made between ‘expert’ and ‘amateur’ (Cronin and Sugimoto 2014; De Rijcke and Rushforth 2015).

The concept of judgment device will provide an additional means of describing researchers’ engagement with the JIF. The sociologist Lucien Karpik (2010) coined the term to describe trusted devices to which buyers in markets for ‘singularities’ delegate when making choices. Singularities are goods that face competition by qualities rather than price (p.39), and are multi-dimensional and incommensurable.^2^ Andrew Abbott (2014) recently picked-up this theme to account for various tools used by individuals and organizations to deal with situations of *excess*. University rankings would be examples of judgment devices, as they facilitate prospective students in ordering their judgments over college choices *hierarchically* (Abbott 2014). This ‘take the best, forget the rest’ formula is more likely to figure in situations where choice is characterized by excess rather than by scarcity (ibid). The appeal of bibliometric indicators is often framed in related terms, with promises to ‘reduce complexity’ being features managers and policymakers find attractive (Cronin and Sugimoto 2014; Woelert 2013). In evaluation contexts journal ranking tools like the JIF help render the prestige from publishing in one journal over another commensurable (Espeland and Stevens 1998), which is appealing in the face of excessive choice, uncertain qualities, and/or the absence of substantive expertise. How mobilizations of the JIF played-out in specific research practices is the issue to which we will now turn.

## Collaboration, Authorship, and the JIF

That scales of problems in modern biomedical knowledge making now necessitates greater interdependencies and expanded forms of collaboration has been well documented by historians and sociologists of science (Shrum et al. 2007). Yet despite ‘increasing incentives to collaborate,’ preservation of the institution of authorship retains the individual as the ‘epistemic subject’ of knowledge production in biomedicine (cf. Knorr-Cetina 1999: 167). Recent neighboring work from sociology of science predicts one of the major impacts of expanding formal evaluation regimes on academic work settings is re-configuring of authority relations *among* scientists, as well with their organizational context, patrons, and stakeholders (Whitley and Gläser 2007; Whitley et al. 2010). Following Whitley (2007), tensions between the need of researchers in fields like biomedicine to collaborate *and* compete may increase as various forms of quality rankings come to ‘intensify the stratification of individual researchers, research teams and employer organisations’ (Whitley 2007: 10). As collaboration is such an important building block of biomedical knowledge, incidences of the JIF in such contexts and its capacity to reconfigure relations among biomedical researchers is of particular interest. How then collaborations were organized and authorship credits distributed, how informants mobilized the JIF as a judgment device in shaping these decisions, and who was able to shape these relations will now be unpacked further.

### Ordering Collaborations in the Laboratory

In our laboratory-based research sites producing a journal article is beyond the competencies of an individual, with collaborations the norm. The researchers followed a rather familiar set of authorship conventions, with the first author in principle the individual who produced the greatest number of figures for a paper, and second authors having contributed fewer. The PI was by convention last author on the paper, which signifies they initiated, facilitated, and lead on the research theme under which the paper marks a contribution. One of the notorious effects these widely followed arrangements give rise to is competition for first author berths. Müller’s (2012) study of Austrian life sciences argues post-doctoral researchers are especially exposed to first-authorship priority struggles, given their positioning at a ‘bottleneck’ between temporary post-doc positions and dwindling numbers of permanent academic openings. We found that PhDs also stood to benefit from high impact first author publications in terms of making them more attractive commodities on the academic job market. Although perhaps not mandatory to finding *some* post-doc position, it was commonly felt that to acquire positions at prestigious laboratories this was essential. We found that PIs in our research sites also faced pressures to produce last authored publications in prestigious journals in order to account for the activities of their labs and departments, to convert publications into grant money, and to continue attracting attention to the laboratory. High impact publications can be advantageous for middle-authors, yet in this kind of authorship system the first and last authors are by common consent always likely to reap the greatest amount of credit. It was no coincidence that technicians were by far the most dismissive set of respondents towards the demands of this reputational economy, as they are seldom credited as first or last authors on publications or directly dependent on these for career progression. For those pursuing a professorial career it is first and last authorship berths (depending on their career phase) which promise the greatest reputational pay-off for the individual; dynamics which we observed across our laboratory-based settings, and we suspect are rather typical of how the JIF feeds into authorship practices for a great number of laboratory-based biomedical research sites.

The institutionalized division of labor in these sites means it is the PI whose name is made visible within the wider peer networks and who is responsible for duties like attracting big grants, hiring staff, identifying promising research topics, retrieving cutting-edge information from conferences, spotting trends in the literature, and so on. The PI’s job is thus primarily office based, whereas junior colleagues are situated much more at the laboratory bench, carrying out the practical embodied labor of experiments along with technicians. In this organizational structure members of the laboratory are ‘elements in [their leader’s] arrangement’ (Knorr-Cetina 1999: 221; Hackett 2005). In one surgical oncology site, one challenge the PI cited was getting part of the PhD population entering the laboratory to meet *his* interests in producing high impact output. In order to do so, the PI had set a minimum requirement of ‘impact points’ each incoming student had to agree to meet before they could submit their thesis.^3^ An important factor in introducing this target came from the translational focus of the laboratory, which hosted researchers with biological *and* clinical training backgrounds. Over half of the PhD students were pursuing careers as surgical specialists in the Netherlands, rather than as academic scientists:

It used to be different, because the bar was set at four publications as a requirement for the PhD. But then we noticed that the [surgical] PhD students were going for minor papers; “As soon as I have these four papers, I can get my PhD, and then I can go into training, or at least I can apply for a training position.” And already, 10 years ago, when we started, we said, “Okay, we have to do this differently, because we’re aiming for quality,” because if you’re not producing quality, you’re not going to get grant money. Nobody’s going to give you a grant if you have four papers in an impact factor one journal, but you may get a grant based on a paper that you published in an impact factor 12 journal or higher, right? And so at that time, we said, “We have to change the requirement for getting the PhD,” and now, we set that bar at 15 impact points. So if you get a paper in an impact factor 15 journal, basically, you’re done. And we’ve really noticed a change in that stimulating people for the quality, and go for that one nice paper.

(PI Interview, Surgical Oncology, Institute B)

Aside from the tight coupling of words like ‘quality’ with the JIF, this shows the PI will only permit those who are like-minded and agree to fulfill *his* ambitions and requirements to enter at this level. Furthermore, it was the PI who was responsible for making decisions about how to distribute resources among the laboratory. Here the JIF was mobilized as a judgment device in evaluating work-in-progress, sometimes influencing whether to continue supporting projects or arms of projects:

Respondent: I just had a discussion with [PhD] on a project that’s never going to be high impact. But then we have the choice; either publish it in a lower journal, or forget about it. And then, of course, we’re also practical and say, “Okay, we have to publish it.”

Interviewer: Okay, yes. So you can decide whether to do more experiments on the basis of whether you think it stands a chance in a higher impact journal.

Respondent: Of course, but then if we stick to [same PhD] as an example, she also has projects that are running really well. And so then, my problem, or something that I have to decide is are we actually going to invest in that project that we don’t think is very high impact, or are we going to try to publish it as it is, in a lower journal, so that she has all the time to work on the projects that are going well, and that do have an interesting set of results?

(PI Interview, Surgical Oncology, Institute B)

The PI thus appears to order judgments hierarchically via the JIF, including how to allocate scarce resources, how much encouragement and attention to give various projects in his laboratory, and how much time to spend co-authoring a paper. Yet rather than recognized as hierarchical commands, the relations of authority and seniority were often framed in laboratory settings in terms of mentorship and guidance. The early-career researchers’ reliance on the PI to mediate the social world of science was exemplified by asymmetries in writing experiences:

Interviewer: Gets it kind of right for the journal?

Respondent: Yes, she [PI] has more experience in publishing and PIs in general [have] more experience to make the message clearer. When you’re a young scientist, you’re a bit crazy and you want to say everything and it makes it a bit more confused. So PIs in general they are really good to say *that* is the main message and [making it] easier to read.

(PhD Interview, Molecular Cell Biology, Institute B)

Authority of laboratory leaders over members is drawn from their ability to mediate between ‘inside’ of the laboratory and ‘outside’ (enjoying greater access with peers, funders, research managers and so on) (Knorr-Cetina 1999: 222, 224; Hackett 2005). Laboratory-based junior members recognized their PI’s positioning as spokesperson for an ‘external context’ was not simply driven by interests in building careers of lab members, but were also responsive towards sets of accountability relations which senior researchers alone must attend (for instance, grant writing and answering to research managers). Our materials suggest that in the laboratory-based biomedical settings PI’s mediations between the external context and members of their laboratory often brought forward and negotiated the JIF as a common interest in collaboration. In these settings then the authority of the laboratory leaders appeared consolidate around the presence of the JIF and the imperative to score highly on its scale.

### Shaping Internal and External Collaborations

By way of contrast we will now turn our attention to the ‘outliers’ in our case: the medical statisticians. The structure of the statistics departments in relation to research was quite idiosyncratic and is worth outlining, as it has consequences for how the JIF fed-into collaborative and authorship relations. Here we focus primarily on Institute B, where there were two ‘tiers’ all staff in the department recognized: researchers and teachers. The ‘researchers’ engaged in their own independent research whereas ‘teachers’ did not. Those who produced their own research were eligible for promotions along the ranks of assistant to full professor, whereas this path was restricted for teachers. One such ‘teacher’ was in fact employed as ‘research support’ staff, whereas two more were assistant professors, hired a number of years before the ‘research active’ members were appointed. Yet these self-professed teachers openly expressed little desire to pursue a research career in the way the ‘research active’ colleagues were doing. In practice the evaluation criteria used for ‘researchers’ and ‘teachers’ differed, with the former evaluated much more in terms of ‘traditional’ indicators (publication numbers, citations, prestige of journals and so on), external funding, and numbers of PhD students. However, both ‘researchers’ and ‘teachers’ were responsible for contributing research output via consulting. Therefore, to describe the forms of collaboration and authorship characterizing knowledge production in this site, it is necessary to attend separately to the production of research output from ‘independent’ and ‘consulting’ research activities.

‘Research active’ members producing their own ‘independent’ publications are engaged in a more individualistic work model compared with the laboratory sites (where there was regular interaction at the laboratory bench, as well as weekly supervisory meetings). The *scales* of their problems did not always necessitate pooling of expertise or very large numbers of collaborators, in the way one readily associates with the ‘big science’ era of biomedicine (cf. Biagioli 2002: 495). Much of the time spent observing PhDs and post-docs in their offices found this activity consisted mostly of them working on their own projects whilst sitting in silence, typing away at their computer workstations. This comparatively individualistic model translated into how authorship for papers got divided. In an associate professor’s emerging research line of biostatistics, decisions to bring in a biologist took the form of an ad hoc arrangement contingent on the perceived complexity of the data set he was working on at that time (Fieldnotes April 3, April 15). The following extract recounts a conversation with a PhD student regarding a manuscript he was preparing to submit as lead author, with his supervisor and a fellow PhD as co-authors. We discuss how typical such an arrangement was of how they divided authorship credits:

We talk about papers at lunch… PhD 1 says of course because X is his supervisor, he will be co-author on his papers. PhD 2 [in the same department] is co-author because they chat all the time and this shapes the work, so she needs to be credited… Again he reiterates the point it is not typical to work with another PhD student unless you have a significant overlap and it ‘makes sense.’

(Fieldnote 17 April)

This explanation of why the manuscript has taken shape in such a way illustrates how this member of the department frames a ‘typical’ collaboration: the role of supervisor entails a legitimate claim to co-authorship, whilst involvement of another PhD though atypical in this instance was worthy of co-authorship credit. Another contrasting aspect of authorship practices with our laboratory-based sites was that top-down pressures for PhDs and post-docs to produce high impact first author publications were much less apparent.

To be clear, the statisticians *did* still produce high impact journal outputs. However, the *means* through which they did so and how these outputs were used to evaluate individuals differed markedly here to our laboratory sites. In their history of oncology clinical trials in the United States, Keating and Cambrosio (2012: 133) remark that medical statisticians have long been used by other sub-fields of medicine as ‘hired-hands’ and consultants. Consulting on external clinical research projects was the primary means through which high impact contributions (as co-authors with clinicians) were achieved. The consultancy was divided ad hoc among all members of the department, including research and teaching staff. This means members of the Department with less resources or inclination to develop core research lines (‘teaching staff’) were also able to make visible contributions to the Department’s ‘research’ output via this authorship model. There is a striking contrast between the dismissal of the JIF as a judgment device in producing independent statistical research, compared with how the indicator gets used in their negotiations as consultants with ‘clients,’ captured in the following fieldnote recording a conversation with a member of ‘teaching’ staff:

Ethnographer: What happens if a paper they consult on gets rejected by journals?

‘Teacher’: Well we normally take the reviewers’ comments into consideration, then slide down the impact factors until we find a venue. This usually works but occasionally things don’t get published… We often can have quite a bit of editorial control about what corrections to make - particularly for our parts of the paper.

(Fieldnote 12 May)

Despite active participation in the work, statisticians were seldom listed as first, last, or even second authors on such papers. This example outlines a form of authority struggle over *which* sub-field of biomedicine gets to identify appropriate journals (the statisticians adapt towards their clients’ demands). A further credibility struggle emanating through their commercial consultancy was in demonstrating the worth of their contribution to those tendering their services (UMC clinicians). This customer-consultant relationship was not always straightforward to execute, especially as receipt of payment often generated assumptions among clinicians that this frees them of obligation to include the statisticians as co-authors. Prima facie this struggle resembles Biagioli’s (2002) observations that growing involvement of private funding sources in the ‘big science’ era brings about an increasing ‘entrepreneurial ethos’ towards authorship (p. 495). In this situation the *credit* rather than *responsibility* function of authorship gets accentuated, with authorship being treated like a ‘trading chip in an economic game’ (Biagioli 2002: 497). The informants’ deployment of *The Vancouver Protocol* as an international standard in biomedical authorship practices was one tactic they stated to negotiate this co-authorship (dis)agreement with collaborators/customers. Members of which biomedical subfield get to decide on order of authorship contributions (or even deny them altogether) which becomes an emerging site of struggle in these ‘mutually beneficial’ collaborative work arrangements. This unorthodox model of pursuing high impact publications came with some difficulties. Nonetheless as their ‘clients’ typically favored ‘aiming high’ in terms of the JIF for their manuscripts, informants reported that consultancy activity accrued large numbers of co-authorships in prestigious titles across various clinical specialties. Despite these conflicts, the statisticians benefitted from this co-authorship arrangement as it meant the department and division were assessed very favorably within the UMC’s annual evaluations, especially when it came to inter-Division comparisons. The Head of Department posited the explanation that such tensions derive from resentment held by ‘customers’ outside their Division who were paying them money and listing them as co-authors, thereby leading the Division to perform outstandingly in UMC evaluations against which the ‘customers’ own divisions would then be unfavorably compared (Fieldnote 15 May). That these particular co-authorship struggles were mediated by the presence of a commercial fee was given weight by our analysis of medical statisticians in *Institute A*. Here the statisticians were financially compensated for their consulting services by the UMC board and no such quarrels over authorship were reported. The imperative to deliver high impact publications thus poses different challenges to these two groups, which are influenced by the organizational provisions in place. These moments underline the different stakes which are at play in the targeting of high impact journals in biomedicine and how different collaboration and authorship practices intersect with this issue – sometimes amicably, other times less so.

A further consequence of statisticians’ practices of obtaining middle-author contributions was that it did not translate very successfully into attracting individual funding from national research councils in the Netherlands, which they claimed typically are interested in evaluating first or last author contributions in high impact titles (Associate professors 1&2 interviews, Institute B). This sense of frustration is relayed in the following explanation of how the associate professor has fared in competing for prestigious public funding at the national level:

Interviewer: Okay, what kind of sources do you look for funding…

Associate Prof: …So yeah the NWO - our national council for scientific research - where you can ask for funding- they don’t have a line or a compartment in which biostatistics naturally fits in with its topics. Actually the only thing you can apply for at NWO is personal grants… But that’s very difficult because, for us, you should be applying within the medical pillars… and in the medical sciences you then have to compete with those guys from the lab who have really five *Science* papers already when they are thirty. We don’t have this [in medical statistics].

This suggests that despite scoring well within UMC evaluations, the statisticians struggle to acquire other forms of external credibility. Here a frequently heard complaint is relayed that there is no venue for their contributions that equates to the brand reputation and impact factor of journals like *Science*. Traditionally statisticians have labored to attract large amounts of funding in medicine, often owing to a perception they are presiding over a ‘method’ rather than ‘subject’ (like ‘cancer’) (Keating and Cambrosio 2012). Although this historical account of (relative) institutional marginality does not cover all the complexities of our statisticians’ situations, it resonates.

The authorship practices of medical statisticians in *Institute B* differed quite markedly from the laboratory-based sciences, whether this meant producing disciplinary contributions as ‘research active’ individuals (where the JIF is seldom forefront in decisions over manuscripts), or contributing to external clinical research projects as consultants (where the JIF is foregrounded, but they cannot acquire the most sought-after authorship berths). How the statisticians’ authorship practices interact with evaluation regimes therefore also appears quite distinct from laboratory-sites. In one moment – annual UMC evaluations – the statistics department appears highly successful by contributing towards their division being a top performer in the UMC. Despite having carved out a niche within the UMC organization, their collaboration and authorship practices come under alternative forms of pressure via A) the commercial exchange of a fee in their consultancy activities, where their clients see them as paid consultants rather than academic partners, thereby often contesting the statisticians’ authorship claims; B) the demands of external funding agencies (on those striving for a conventional academic research career). In this latter ‘game’ of individual grant writing they appear to perform relatively poorly compared with the laboratory-based sites, as external legitimacy in this context seems very tightly associated with the ability to produce first and last author publications in high impact titles. This reiterates our point that although the JIF is an important concern across each of the sites, how it intersects with authorship and collaboration aspects of knowledge production at different levels of authority relations appears to vary considerably. ‘Research active’ statisticians usually worked in smaller teams of co-authors than those in the laboratory-based sites and targeted journals with ‘relevant audiences’ and prestige in their peer group. This appeared to shape the kinds of interactions observed around the JIF, which was mentioned in respect to their own independent research less frequently, always with qualifications, and even with some irony. This irony was also explained to us by the fact that most of these researchers have a background in mathematics; they tended to be keenly aware of technical and mathematical limitations in the calculation and application of seemingly straightforward indicators such as the JIF and the H-index (Assistant professor interview, Institute A).

Multiple demands for high impact outputs appeared to reinforce patterns of authority based on interdependence and seniority in the laboratory-based sites. Müller (2014) recently reported on increasing forms of instrumentalism between post-doctoral researchers and students whom they mentor in life science settings. For Müller possibilities for more pastoral models of supervision and co-authoring are eroded in favor of instrumental norms and behaviors based on exchange (as supervision is traded for getting one’s name on a paper). In situations where first and last author publications are especially de rigueur and a homogenous indicator persists, instrumental relations towards authorship and journal targeting of this kind are arguably likely to intensify (ibid). We would add that although there were some signs that the JIF and the stakes associated with it can be seen to intensify such instrumental relations around publication activities between senior and junior figures (not just at the level of post-docs and students), our findings on this point are tentative and require follow-up research.

## The JIF and Targeting of Journals: Scientists’ Folk Theories

The prestige associated with publishing in reputable journals acts as a powerful incentive that scientists will almost always consider in the course of their work. Prestige here can be understood as a product of knowledge work which gets captured and mobilized as an ‘exchange good’ by academic scientists (Stephan 2012). But what reputational criteria do biomedical scientists draw on when targeting their work towards particular titles in the course of their research? Are such considerations becoming ever more tightly coupled with the JIF, or do other considerations still factor? The primary empirical materials in this section are taken from observations of supervisory meetings between senior and junior researchers in the process of preparing together manuscripts for submissions to journals. These occasions are useful entrance points not only to illustrate how JIF functions as a ‘judgment device’ in mundane research settings, but also to compare folk theories about the indicator. We show these respective folk theories are consequential insofar as they shape researchers’ A) sense-making; B) potential actions they sought to take, and, importantly, C) the knowledge they create.

### Grading for Novelty and Quality

Surgical oncologists usually expressed little ambivalence regarding the reliability (and indeed validity) of JIF as a judgment device in their work. During meetings and interviews a consistent folk theory was projected linking impact factor to the *demands for novelty and competitiveness from a given journal*. Oncologists reasoned the novelty demanded by journal gatekeepers rested on the identification of a novel biological mechanism linked to cancer development in cells. In this ‘mission-oriented’ epistemic culture this identification is valued because it harbors greater promises for clinical translation. Contributions to knowledge that fall short of the mechanism threshold get counted as ‘descriptive,’ with little prospect for being taken up into subsequent translational, proof-of-principle studies. Descriptive contributions are found only in the lower impact journals that are considered less reputable. The following moment begins with a supervisory meeting between a surgical oncology PI, his PhD student, and a post-doc, who are discussing a manuscript they were preparing for publication together. The following exchange exemplifies how oncologists mobilized this folk theory in the course of decision-making about where to target the manuscript:

[PI] goes to computer. PI: Any alternatives? Any journals?

… PhD: Hmm maybe *Journal C*. They are similar in impact right?

Post-doc: Yeah seven-ish. It’s difficult because some papers are descriptive and some have mechanism. So for this paper it could actually go one step higher than *Journal C* because you’re going a bit beyond description. They also have priority reports in *Journal B*.

PI: *Journal D* also have [sic] very fast publishing periods from date of submission - if they like it of course.

(Fieldnote 22 July)

In this particular instance potential for the manuscript to ‘go beyond description’ prompts the post-doc to suggest they disqualify (initially) *Journal C* and go ‘one step higher.’ This correlation between the JIF and a journal’s novelty requirements has clear links to why some titles are more reputable than others. However, ‘novelty’ is not the only requirement of the top-tier impact journals in their field: some respondents argued the amount of rigor and labor required to show beyond doubt the strength of one’s claim to have identified a given mechanism (‘quality’), correlate reliably with the impact score of journals in their field. The promise of capturing higher prestige also makes high impact titles more competitive and the journals are able in turn to reject articles that do not prove mechanisms. This sense of tighter editorial policing was evident later in the conversation:

PI: I know I said *Journal A* but I don’t know if it’s good now. I reviewed a couple of papers for them recently and it’s an unbelievable amount of work expected of authors.

PhD: Yes it’s high impact.

(Fieldnote 22 July)

The JIF emerges here as a judgment device for betting on the *likelihood of rejection*. This linking of journal reputations to impact factors in this sub-field rests on assumptions about how papers are generally cited among their peers. The PI states:

The thing is we published data in journals with 2 or 3 impact before, they may not be bad journals but you are lucky if it gets cited ten times over the years. So what is the point in sending it there?

(Fieldnote 8 July)

The reasoning here is that articles appearing in high impact journals generally attract larger citation numbers *precisely because they are published in high impact journals*. This ‘Matthew Effect’ appears to suggest the very citing of articles among their peers is informed hierarchically via the impact factor score of the journal in which they are published. The above extracts show oncologists’ folk theories of the JIF have important implications for how they evaluate their own work-in-progress (is it ‘good enough’ for the higher impact title, is the manuscript ‘wasted’ if it goes to this lower impact title and gets accepted straight away, can we get it higher than it ‘deserves’?). This crucially shapes decisions about whether or not to continue working on the manuscript, for instance, if another set of experiments and figures are needed in order for it to ‘stand a chance’ at high impact title, or whether to settle for lower impact. It suggests then that JIF-talk is more than simply informal publication-talk through which biomedical researchers typically evaluate colleagues and peers (contra Knorr-Cetina 1999: 222–224). As with the surgical oncologists, molecular cell biologists in our study often used argumentative registers about novelty and quality/rigor of contributions demanded by the top journals to explain their theories of the indicator. The following moment intersects a meeting between a PI and two PhD students discussing a manuscript they were working on. The PI has posed the question to the students of what the manuscript is ‘worth’ in terms of JIF points. One of the students provided a joking comment that they would send it to the prestigious *Nature*. This form of joke was common across our material when observing scientists discuss manuscript destinations. The response of the professor – also typical across our material – was to laugh along but also remind the student to ‘be realistic.’ Such exchanges suggest the JIF is consistently taken as a reliable judgment device to bet on likely rejection rates of different journals. Once the joke has passed, the informants move to discussing novelty:

PhD 1: I think it is new…it is specific to mammalian cells… maybe *Journal A*. I don’t know.

Prof: What is new is that we provide insights into an important pathway - that is the underlying message. We identify the specific pathway mediated by this machinery.

PhD 1: Let’s go for a seven. If we really manage to describe the marker then that *really* is new.

PhD 2: Well we don’t know all the proteins yet.

Prof: We can only talk about what we show here.

(Fieldnote 30 June)

Their scoring of the manuscript revolves initially around the novelty of the figures they have produced and have printed out on the A4 paper in front of them, with the professor adopting a position of a skeptical reader who the PhDs have to persuade about the value of the paper (measured in terms of impact score). Here then, as with the surgical oncologists, the cell biologists draw on assumptions that the JIF correlates more-or-less to the novelty of contributions in a given journal.

### Ambivalence

Thus far interactions in the two laboratory settings appear to posit the JIF as a reliable indicator for estimating novelty and quality of a particular title and thereby its likely levels of editorial rigor and rates of rejection. However, later in the same discussion among molecular biologists ambivalence was expressed when part of the common theorizing about the JIF was explicitly queried by one of the PhDs:

R: I always see [studies] in *Cell* [a high impact journal] and honestly they are not that good, I cannot see it as any different from our papers…

At which point the professor responds:

Prof: It is the quality of the data that makes the difference. The message now is always the impact factor.

(Fieldnote 30 June)

The theory about the ‘quality’ of figures that top-tier impact journals demand of published papers was mobilized by the professor to repair the JIF as a common matter of importance in the conversation. This demonstrates how labor is needed to (re)incorporate the JIF into the social and material realities of doing research, particularly if fault lines emerge in respondents’ theorizing of the JIF. Interestingly, questions of how desirable it was to obtain a higher impact score and justifications for why it was important were regularly raised by the informants in this molecular biology group, in marked contrast with the surgical oncologists in our study, where widely discussed issues regarding general shortcomings or criticisms of the indicator were largely absent.

When compared to cancer-related sub-fields, medical statistics journals do not generally carry high impact scores. This makes for a non-standard story of how ‘research active’ members from this site incorporated the JIF into their work. Those members who are evaluated in terms of their research contributions as individuals have an ambivalent relationship with the indicator. The following account from a PhD was typical of statisticians’ responses:

I: And do you look at things like the JIF?

PhD: Impact factor. Well it’s a bit difficult especially in a statistics field where most of the journals don’t really have high impact factors. For instance, in a journal like *Journal A*, it is really a good journal in medical statistics, but it will only have like two-point-something [JIF score]. But if you go to a general epidemiology journal, then two-point-something is a very low journal. They will usually aim for something like seven or ten impact factor. But for us two-point-something is really high.

(PhD interview, statistics, Institute B)

On the one hand, the PhD is aware of the disparity between impact scores of journals between medical sub-fields as even those neighboring their own (epidemiology) publish in much higher scoring titles. Yet this does not lead to complete dismissal of the indicator when identifying journals *within* the scope of their statistical specialty, but only a claim that it is misleading when comparing *between* sub-fields. This epistemological argument was typical of theorizing about the JIF across statistics sites. The attributed shortcomings were palpable in one instance where the PhD and her associate professor supervisor had sent a co-authored paper to a multi-disciplinary journal as a ‘last resort,’ having been rejected from six statistical journals they had considered more prestigious. Ironically, this ‘last resort’ had almost double the impact score as the statistics journals that had rejected the manuscript. In circumstances where they sought recognition from publishing in statistical titles additional criteria were brought forward like the journal’s ‘fit’ with their given topic, relevance of its readership, and likelihood of accruing future citations. This suggests a heavily qualified folk theory about the reliability of the JIF in relation to prestige of statistical journals: it does not convey well the novelty or contribution of a particular article to those outside the sub-field.

The statisticians’ example demonstrates that widespread propensity to promote impact scores throughout biomedicine does not always sync with how scientists in certain sub-cultures of biomedicine attribute reputation in targeting journals. Yet it is also notable that despite all these subtle gradations in the statisticians’ theorizing, the JIF was still present in their knowledge making process. As a de facto performance standard, the indicator was much more readily incorporated as a judgment device into laboratory scientists’ decision-making around actions to be taken on manuscripts. Here the JIF appears to be much more tightly correlated with aspects of novelty and quality journals demand of submitted manuscripts. Of course, it is highly likely that informants’ folk theories were themselves responsive towards folk theories in wider circulation across the social world of biomedicine, which acquire a kind of self-fulfilling effect (Rip 2006). A consequence for work in these laboratory settings is that JIF-considerations seemed to mediate and sometimes eclipse judgments of the kind statisticians were making about journals, like readership particular titles typically attract, or the reputations a title carries among peers. However, the uses of the JIF are multi-dimensional and laboratory-based scientists do, of course, still make qualitative judgments about where to send manuscripts (c.f. Karpik 2010). Our findings here lead us to form the tentative suggestion that when issues like novelty and priority of findings become ever more synonymous with the JIF, then aspects of knowledge production like journal targeting will become increasingly attuned and attentive towards this pre-defined indicator.

## Conclusion

At present a number of folk theories percolate in the world of biomedical science about the relative importance and role of the JIF. Whilst these public statements characterize the indicator as part of ‘the culture’ of biomedicine at large (e.g. Brown 2007; Misteli 2013), they stop short of stating that it has been incorporated into biomedicine’s ‘epistemic culture(s).’ Contrary to these moves, we have analyzed here two building blocks of biomedical knowledge production the JIF latches onto as a judgment device: A) collaboration and authorship practices; and B) assessing work-in-progress manuscripts and how novelty of work and reputation of journals are evaluated. Taken together, our findings suggest this indicator should not be dismissed as mere idle ‘publication talk,’ or as floating in some external ‘cultural’ realm separated from the ‘serious business’ of knowledge making. Likewise statements of discontent tend to implicate the entire field of biomedicine as captured by the JIF, yet the comparative scope of our findings raises the question of which sub-cultures are more-or-less attentive to JIF-considerations and how might this differ. A more general problem with many received criticisms of the indicator is they primarily focus on epistemological properties, suggesting this is the level on which researchers typically engage the indicator. It was possible, however, for informants to be aware of epistemological limitations, whilst simultaneously recognizing an article in a high impact title was ‘the ticket’ needed to secure a grant or job position. Thus even if informants do not necessarily mobilize the JIF primarily as an epistemological device to calculate quality, this does not mean there was complete absence of sociological theorizing or blanket ignorance about the indicator. For this reason we feel ambivalent about statements coming from scientometricians that the JIF ‘misleads.’ By limiting indicator uses to questions of validity, movements like DORA also assume displacing the JIF for ‘better’ (i.e. more valid) indicators would necessarily give rise to better evaluation practices. Again this ‘modest proposal’ appears to be borne out of the assumption that the JIF is external of ‘core’ research practices. Our findings suggest that in calling for researchers to ‘drop’ the JIF, DORA is actually calling for transformations in how biomedical knowledge is manufactured. Although calls for better indicators are difficult to refute in principle, our findings serve to remind that in research practice ‘better epistemological indicators’ will always generate their own constitutive effects (Dahler-Larsen 2013).

Whilst we recognize the thrust of certain arguments concerning homogenization of performance evaluation around the JIF in biomedicine, the ethnographic material we put forward about scientists’ work patterns around the indicator can help qualify such assumptions, including also those found in critical social science domains. Treating quantitative indicators as merely receptacles for top-down control over academics risks downplaying how indicators acquire additional meanings through their uses (Dahler-Larsen 2013). In some of our biomedical sites indicator uses may be said to ‘conform to types’ set-out in critical studies, yet in other moments they appear confounding. Even in settings where a single indicator – like the JIF – appeared an obligatory concern to all, there are still other forms of information and indicators being filtered through the JIF, which go beyond generalized profiles of researcher ‘responses’ or ‘perceptions.’ Rather than undermining then the claim that the JIF now dominates biomedicine, our study textures these accounts and evokes detailed empirical materials as the basis for further reflection and theoretical enrichment of its presence within this important field of research. Our employment of the term ‘judgment device’ to conceptualize use of the JIF – whilst insightful – requires qualification. Although the indicator appears to meet a number of abstracted characteristics, Karpik’s (2010) original argument that judgment devices enjoy widespread trust among users ought to be tempered by consideration that respondents here were knowledgeable about some widely-discussed criticisms of the JIF. Here then it is instructive to distinguish between the JIF’s assumed reliability in contexts of use (which was strong) and validity as a measure of quality in science (which respondents conceded was open to questioning). Detailed empirical examinations of indicators in other epistemic and organizational settings would enrich theories of indicator use in sociological studies of science and higher education, and inform ongoing normative and political debates surrounding the ‘crisis’ of quantitative indicators in science. We hope our findings can provide a platform for further research into the presence of the JIF in different regional and epistemic contexts, in order that the implications of this controversial indicator might be more fully considered.

## Footnotes

1 This institutional and epistemic setting is important because historically biomedical research evaluation has been particularly responsive towards quantification and standardization, with the earliest Dutch science policy document on assessing the sciences focusing on the medical sciences (RAWB 1983). The RAWB report was promoted as a model for priority setting in research, and was the first to overthrow internal peer review as the sole source for assessment by including citation analysis (Wouters 1999). Since then, the use of performance indicators for medical research is increasingly favored over peer review by policymakers in the Netherlands (IRMEC 2005; VWS 2006).

2 The commercial imperatives of competing for market share through judgment devices (Karpik 2010: 51–52) can be extended to Thomson Reuters’ promotion of the JIF.

3 In Dutch biomedicine it is a customary format for PhD theses to consist of published journal articles, rather than in monograph form.
